# Machine learning and earthquake forecasting—next steps

**DOI:** 10.1038/s41467-021-24952-6

**Published:** 2021-08-06

**Authors:** Gregory C. Beroza, Margarita Segou, S. Mostafa Mousavi

**Affiliations:** 1 Department of Geophysics, Stanford University, Stanford, CA USA; 2 British Geological Survey, Research Avenue South, Lyell Centre, Edinburgh, UK

**Keywords:** Natural hazards, Seismology

## Abstract

A new generation of earthquake catalogs developed through supervised machine-learning illuminates earthquake activity with unprecedented detail. Application of unsupervised machine learning to analyze the more complete expression of seismicity in these catalogs may be the fastest route to improving earthquake forecasting.

The past 5 years have seen a rapidly accelerating effort in applying machine learning to seismological problems. The serial components of earthquake monitoring workflows include: detection, arrival time measurement, phase association, location, and characterization. All of these tasks have seen rapid progress due to effective implementation of machine-learning approaches. They have proven opportune targets for machine learning in seismology mainly due to the large, labeled data sets, which are often publicly available, and that were constructed through decades of dedicated work by skilled analysts. These are the essential ingredient for building complex supervised models. Progress has been realized in research mode to analyze the details of seismicity well after the earthquakes being studied have occurred, and machine-learning techniques are poised to be implemented in operational mode for real-time monitoring. We will soon have a next generation of earthquake catalogs that contain much more information. How much more? These more complete catalogs typically feature at least a factor of ten more earthquakes (Fig. 1) and provide a higher-resolution picture of seismically active faults.

**Fig. 1:**
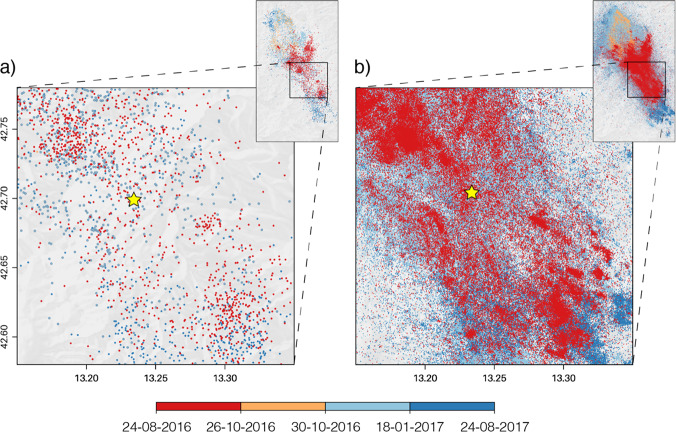
A year of seismicity in the epicentral area of the 2016 *M* = 6.0 Amatrice earthquake (star) in Italy color coded by time of occurrence. **a** Real-time catalog, available at http://cnt.rm.ingv.it/ and (**b**) machine-learning catalog^16^ are shown for event magnitudes above their respective magnitude of completeness^12,16^ Mc = 2.2 and Mc = 0.5.

This next generation of earthquake catalogs will not be the single, static objects seismologists are accustomed to working with. For example, less than 2 years after the 2019 Ridgecrest, California earthquake sequence there already exist four next-generation catalogs, each of which were developed with different enhanced detection techniques. Now, and in the future, this will be the norm, and earthquake catalogs will be updated and improved—potentially dramatically—with time. Second-generation deep learning models^1^ that are specifically designed based on earthquake signal characteristics and that mimic the manual processing by analysts, can lead to performance increases beyond those offered by earlier models that adapted neural network architectures from other fields. Those interested in using earthquake catalogs for forecasting can anticipate a shifting landscape with continuing improvements.

While these improvements are impressive, the value of the extra information they provide is less clear. What will we learn about earthquake behavior from these deeper catalogs and how might it improve the prospects for the stubbornly difficult problem of earthquake forecasting?

Short-term deterministic earthquake prediction remains elusive and is perhaps impossible; however, probabilistic earthquake forecasting is another matter. It remains the subject of focused and sustained attention and it informs earthquake hazard characterization^2^ and thus both policy and earthquake risk reduction. A key assumption is that what we learn from the newly uncovered small earthquakes in AI-based catalogs, will inform earthquake forecasting for events of all magnitudes. The observed scale invariance of earthquake behavior suggests this is a reasonable expectation.

Empirical seismological relationships have played a key role in the development of earthquake forecasting. These include Omori’s law^3^ that describes the temporal decay of aftershock rate, the magnitude-frequency distribution, with the b-value describing the relative numbers of small vs. large earthquakes^4^, and the Epidemic Type Aftershock Sequence (ETAS) model^5^ in which earthquakes are treated as a self-exciting process governed by Omori’s law for their frequency of occurrence and Gutenberg–Richter statistics for their magnitude. These empirical laws continue to prove their utility. Just in the past few years, the time dependence of the b-value has been used to try to anticipate the likelihood of large earthquakes during an ongoing earthquake sequence^6^ and the ETAS model has been improved to better anticipate future large events^7^. So it appears that there is value in applying these longstanding relationships to improved earthquake catalogs, but our opinion is that much more needs to be done.

The relationships cited above date from 127, 77, and 33 years ago. The oldest of them, Omori’s Law, was developed based on felt reports without the benefit of instrumental measurements. We suggest that a fresh approach using more powerful techniques is warranted. Earthquake catalogs are complex, high-dimensional objects and as Fig. 1 makes clear, that is even more true for the deeper catalogs that are being developed through machine learning. Their high dimensionality makes them challenging for seismologists to explore, and the conventional approaches noted above seem unlikely to be taking advantage of the wealth of new information available in the new generation of deeper catalogs. We suggest that, having first enabled the development of these catalogs, the statistical-learning techniques of data science are now poised to play an important role in uncovering new relationships within them. The obvious next step is to apply the techniques of machine learning in discovery mode^8^ to discern new relationships encoded in the seismicity.

There are tantalizing indications that such an approach may lead to new insights. In double-direct-shear experiments, background signals that were thought to be uninformative random noise have instead been shown to encode information on the state of friction and the eventual time of failure of faults in a laboratory setting^9^. Well-controlled laboratory analogs to faults lack the geologic complexity of the Earth, yet, weak natural background vibrations of a similar sort, that again were thought to be random noise, have been shown to embody information that can be used to predict the onset time of slow slip events in the Cascadia subduction zone^10^. Finally, unsupervised deep learning, in which algorithms are used to discern patterns in data without the benefit of prior labels, applied to seismic waveform data uncovered precursory signals preceding the large and damaging 2017 landslide and tsunami in Greenland^11^.

These examples are compelling but come with the caveat that they are not representative of the typical fast rupture velocity earthquakes on tectonic faults that are of societal concern. For such earthquakes, however, there are also indications from state-of-the-art forecasting approaches that next-generation earthquake catalogs may contain information that will lead to progress. Physics-based forecasting models, which account for changes in the Coulomb failure stress due to antecedent earthquakes that favor the occurrence of subsequent earthquakes, have shown increasing skill such that they are competitive with, and are beginning to outperform, statistical models. Coulomb failure models benefit particularly from deeper catalogs because they include many more small magnitude earthquakes. These small earthquakes add predictive power through their secondary triggering effects tracking the evolution of the fine-scale stress field that ultimately controls earthquake nucleation in foreshock and aftershock sequences. They can also be used to define the emerging active structures that comprise fault networks and by doing so clarify the relevant components of stress that would act to trigger earthquakes^12^. Secondary triggering and background stress heterogeneity were shown to improve stress triggering models^13^ but were most effective when they incorporated near‐real‐time aftershock data from the sequence as it unfolded^14^. We note that there is no reason why more complete earthquake catalogs, developed with pre-trained neural network models, cannot be created in real time as an earthquake sequence unfolds. Finally, despite the disappointing history of the search for precursors, due diligence requires that seismologists consider the pursuit of signals that might be precursory.

We conclude that it is now possible to image the activity on active fault systems with unprecedented spatial resolution. This will enable experimentation with familiar hypotheses and enable the formulation of new hypotheses. It seems certain that the underlying processes that drive earthquake occurrence are encoded in this next generation of earthquake catalogs, but we may not find them unless we put new effort into searching for them. Unsupervised learning methods^15^ are particularly well-suited tool for that effort.
